# Impact of Blood Type, Functional Polymorphism (T-1676C) of the *COX-1* Gene Promoter and Clinical Factors on the Development of Peptic Ulcer during Cardiovascular Prophylaxis with Low-Dose Aspirin

**DOI:** 10.1155/2014/616018

**Published:** 2014-08-27

**Authors:** Pin-Yao Wang, Hsiu-Ping Chen, Angela Chen, Feng-Woei Tsay, Kwok-Hung Lai, Sung-Shuo Kao, Wen-Chi Chen, Chao-Hung Kuo, Nan-Jing Peng, Hui-Hwa Tseng, Ping-I Hsu

**Affiliations:** ^1^Institute of Biomedical Sciences, National Sun Yat-Sen University, Kaohsiung 804, Taiwan; ^2^Division of Gastroenterology, Department of Internal Medicine, Kaohsiung Veterans General Hospital, 386 Ta Chung 1st Road, Kaohsiung 813, Taiwan; ^3^School of Medicine, National Yang-Ming University, Taipei 112, Taiwan; ^4^Department of Chemistry, College of Science, National Kaohsiung Normal University, Kaohsiung 802, Taiwan; ^5^Division of Gastroenterology, Department of Internal Medicine, Kaohsiung Medical University Hospital, Kaohsiung 807, Taiwan; ^6^Department of Nuclear Medicine, Kaohsiung Veterans General Hospital and National Yang-Ming University, Kaohsiung 813, Taiwan; ^7^Department of Pathology, Kaohsiung Veterans General Hospital, Kaohsiung 813, Taiwan

## Abstract

*Aims*. To investigate the impact of blood type, functional polymorphism (T-1676C) of the *COX-1* gene promoter, and clinical factors on the development of peptic ulcer during cardiovascular prophylaxis with low-dose aspirin.* Methods*. In a case-control study including 111 low-dose aspirin users with peptic ulcers and 109 controls (asymptomatic aspirin users), the polymorphism (T-1676C) of the *COX-1* gene promoter was genotyped, and blood type, *H pylori* status, and clinical factors were assessed.* Results*. Univariate analysis showed no significant differences in genotype frequencies of the *COX-1* gene at position -1676 between the peptic ulcer group and control group. Multivariate analysis revealed that blood type O, advanced age, history of peptic ulcer, and concomitant use of NSAID were the independent risk factors for the development of peptic ulcer with the odds ratios of the 2.1, 3.1, 27.6, and 2.9, respectively.* Conclusion*. The C-1676T polymorphism in the *COX-1* gene promoter is not a risk factor for ulcer formation during treatment with low-dose aspirin. Blood type O, advanced age, history of peptic ulcer, and concomitant use of NSAID are of independent significance in predicting peptic ulcer development during treatment with low-dose aspirin.

## 1. Introduction

Low-dose aspirin (75–325 mg/day) is widely used in the prevention of myocardial infarction or ischemic stroke [1]. Besides the patients requiring secondary prevention of cardiovascular events, the American Heart Association recommends prophylactic aspirin for the subjects who have a 10-year cardiovascular risk equal to or more than 10% [2]. Currently, approximately 36% of the adult US population—more than 50 million people—is estimated to take aspirin regularly for cardiovascular disease prevention [3]. However, due to its inhibition of prostaglandin synthesis, direct cytotoxicity, and microvascular injury, aspirin is associated with upper gastrointestinal side effects, which range from mild dyspepsia to life-threatening bleeding and perforation from peptic ulcers [4]. Even at doses as low as 10 mg, aspirin has been shown to cause gastrointestinal damage and is associated with a greater risk of gastroduodenal ulcers and life-threatening ulcer complications [5].

Previous studies revealed that a history of ulcer, advanced age (>70 years), concomitant use of nonsteroidal anti-inflammatory drugs (NSAIDs), use of dual antiplatelet therapy,* Helicobacter pylori *(*H. pylori*) infection, and history of alcohol abuse were risk factors for gastroduodenal ulcer in low-dose aspirin users [6, 7]. However, the genetic risk factors for the development of peptic ulcer in aspirin users remain unclear. A recent 35-year cohort study from Sweden also showed that individuals with blood group O had a higher risk of peptic ulcer development than those with other blood groups and blood group A was associated with the risk of developing gastric cancer [8]. Nonetheless, whether blood group O is also a risk factor for ulcer development in low-dose aspirin users remains to be clarified.

Cyclooxygenase-1 (COX-1) is a constitutively expressed enzyme that generates prostaglandins for gastrointestinal integrity. Aspirin can inhibit COX-1 enzyme and decrease prostaglandin synthesis of gastrointestinal tract. Recently, functional polymorphism (T-1676C) in the* COX-1* gene promoter has been identified and was reported to alter putative transcription factor (GATA-1, CdxA) binding sites [9, 10]. Arisawa et al. revealed that the number of -1676T alleles of the* COX-1* gene promoter was a significant risk factor for the NSAID-induced ulcer in Japan [11]. However, whether the* COX-1* genetic polymorphism also plays an important role in the development of peptic ulcer in low-dose aspirin users remains unclear.

The aim of this case-control study was to investigate the clinical risk factors and genetic markers for the development of peptic ulcer during cardiovascular prophylaxis with low-dose aspirin.

## 2. Patients and Methods

### 2.1. Patients

A total of 111 consecutive low-dose (75–325 mg/day) aspirin users with peptic ulcer, who attended the Kaohsiung Veterans General Hospital or Kaohsiung Medical University Hospital, were included in this study. The reason for choosing this dosage range of aspirin is because the common dose of aspirin used for the prevention of cardiovascular diseases varies between 75 and 325 mg daily [1–3]. The diagnosis of peptic ulcer was confirmed by endoscopic examination. One hundred and nine consecutive asymptomatic low-dose aspirin users who underwent upper gastrointestinal endoscopy for endoscopic surveillance and had normal endoscopic appearance or gastritis only served as controls. The exclusion criteria included (1) consumption of proton pump inhibitor (PPI) or histamine-2 receptor antagonist within 2 weeks before endoscopy, (2) coexistence of gastrointestinal malignancies, (3) serious medical illness (e.g., decompensated liver cirrhosis, uremia, septic shock, acute stroke, and acute myocardial infection within 2 weeks), and (4) receiving low-dose aspirin less than 2 weeks. The study was approved by the Medical Research Committee of the Kaohsiung Veterans General Hospital and Kaohsiung Medical University Hospital. All patients and controls gave informed consent.

### 2.2. Methods

Endoscopies were performed with the Olympus GIF XV10 and GIF XQ200 (Olympus Corp., Tokyo, Japan). During endoscopy, biopsies over antrum and body were performed for rapid urease test (with one specimen from the antrum and another one from the body). The rapid urease test was performed according to our previous studies [12]. Rapid urease tests were assessed by a technician, blind as to the endoscopic features. The diagnosis of* H. pylori* infection was based on the result of rapid urease test. An ulcer was defined as a circumscribed mucosal break 3 mm or more in diameter, with a well-defined ulcer crater in the stomach or duodenum [13]. The size of ulceration was measured by opening a pair of biopsy forceps of known span in front of the ulcer.

Before endoscopy, venous blood was drawn for* COX-1* genotyping. To assess the significance of clinical characteristics, the following data were recorded for each patient: age, sex, blood type, smoking, alcohol consumption, coffee consumption, tea consumption, previous history of peptic ulcer, and use of thienopyridine, coumadin, steroid, or NSAIDs within 2 weeks prior to endoscopy. Coffee or tea consumption was defined as drinking 1 cup or more per day.

#### 2.2.1. *COX-1 *Genotyping

Genomic DNA was extracted from 3 mL of whole blood by the use of a QIAamp DNA Extraction Mini Kit (QIAGEN Inc., Valencia, CA). The* COX-1* genotype (T-1676C) was determined using the restriction fragment length polymorphism (RFLP). The primer set (COX-1F, 5′-TGGACCAGTCCTCAGAGACC-3′ and COX-1R, 5′-CCCATC AAGTCACCACACCT-3′) [14] was used to amplify DNA fragments, of which the fragments of 243 base pairs were obtained. The PCR reactions were performed using 2.5 U of Taq DNA polymerase together with the corresponding Taq buffer supplemented with 2 mM MgCl_2_ (Invitrogen, Life Technologies, California, USA), 2.5 mM each of the 4 deoxynucleotide triphosphates (dNTP, 100 mM, Amersham, UK), 0.5 M of each amplification primer, and 40–100 ng of genomic DNA mixed up to a final volume of 25 *μ*L. The PCR analyses were run on a PCR thermal cycler (model 2400, Perkin Elmer, Foster City, CA) with the following profile: 5 min of denaturation at 94°C, followed by 35 cycles of 30 s at 94°C, 30 s at 64°C, and 60 s at 72°C, with a final elongation at 72°C for 7 min. The amplified DNA fragments (243 base pairs) contained two* TspR* I restriction sites. After* TspR* I digestion, fragments with172 base pairs and 71 base pairs were observed for genotype TT; fragments with 172 base pairs, 88 base pairs, 84 base pairs, and 71 base pairs were observed for genotype TC; and fragments with 88 base pairs, 84 base pairs, and 71 base pairs were observed for genotype CC. Then the PCR products were digested by* TspR* I restriction enzymes in a reaction mixture (20 *μ*L) containing PCR products (15 *μ*L), 1X Buffer 4, 1X BSA, and* TspR* I (0.5 U/*μ*L) at 65°C for 16 hours. The digested fragments were separated and analyzed by electrophoresis using 3% agarose gel. Genotypes of* COX-1 *gene promoter were classified into the following three groups: T/T, T/C, and C/C.

#### 2.2.2. Statistical Analysis

Statistical evaluations were performed using the SPSS/Windows computer software package (Chicago, IL, USA). Two-sample* t*-tests were used to compare the mean values of the variables considered continuous in the peptic ulcer patients and controls. The chi-square test with or without Yate's correction for continuity and Fisher's exact test when appropriate were applied to analyze the categorized variables. Differences were considered to be significant at *P* < 0.05. A stepwise forward conditional method was used to identify the independent risk factors for the development of peptic ulcer in low-dose aspirin users. The studied variables included the following: age (<60 or ≥60 years), sex, blood type (O type or non-O type), history of smoking (<1 pack/week or ≥1 pack/week), history of alcohol consumption (<80 g/day or ≥80 g/day),* H. pylori *status (presence or absence), and genotype of* COX-1* gene promoter.

According to a previous study [11], the frequency of -1676T allele in the* COX-1* gene promoter in healthy subjects was 47%. We estimated that a 20% difference in the allele frequency could be present in low-dose aspirin users with peptic ulcer. Based on this assumption, 93 subjects had to be studied in each group to yield a statistical power of 0.80 and an *α* value of 0.05.

## 3. Results 

### 3.1. Clinical Characteristics of Aspirin Users with or without Peptic Ulcer

Two hundred and twenty aspirin users (126 from the Kaohsiung Veterans General Hospital and 94 from the Kaohsiung Medical University Hospital) were recruited for the study. They included 111 subjects with peptic ulcer disease (gastric ulcer: *n* = 90; duodenal ulcer: *n* = 10; both gastric and duodenal ulcers: *n* = 11) and 114 controls.  Table 1 shows the demographic characteristics of peptic ulcer subjects and controls. The subjects with peptic ulcer were significantly older than the controls (67.0 ± 12.1 versus 72.0 ± 10.3; *P* = 0.001). In addition, the rates of peptic ulcer history and the use of NSAID of peptic ulcer subjects were significantly higher than the controls (50.5% versus 3.7% and 16.2% versus 6.4%, resp.; *P* = 0.000 and *P* = 0.022, resp.). However, the peptic ulcer group had a lower rate of coffee drinking than the control group (9.9% versus 22.2%; *P* = 0.033).* H. pylori* infection was detected in 26.2% of peptic ulcer subjects and in 47.5% of the controls. The infection rate was significantly lower in the peptic ulcer group than in the control group (*P* = 0.005). The two groups were similar with respect to gender, tea consumption, alcohol consumption, dual antiplatelet therapy with thienopyridine, and use of steroids.

### 3.2. Genetic Factors Related to Peptic Ulcer Development in Aspirin Users

Table 2 shows the blood group and genotype polymorphisms at position -1676 of the* COX-1* gene promoter in aspirin users with and without peptic ulcer. The distributions of blood groups between aspirin users with and without peptic ulcer were different (*P* = 0.047). Peptic ulcer group had a higher rate of blood type O than the control group (45.9% versus 29.4%, resp.). The T/T, C/T, and C/C genotypes at position -1676 of the* COX-1* gene promoter in aspirin users with peptic ulcer were 24.7%, 66.3%, and 9.0%, respectively. No significant differences in genotype frequencies of the* COX-1* gene were found between the peptic ulcer group and control group (*P* = 0.245).

#### 3.2.1. Independent Risk Factors for the Development of Peptic Ulcer in Aspirin Users

A stepwise forward logistic regression analysis for clinical and genetic variables was performed to search for the independent risk factors of peptic ulcer development in low-dose aspirin users. Multivariate analysis indicated that the advanced age, history of peptic ulcer, use of NSAID, and blood type O were the independent risk factors for the development of peptic ulcer (Table 3). The odds ratios of the four parameters were 3.1, 27.6, 2.9, and 2.1, respectively (95% confidence intervals, 1.4–8.6, 8.9–85.5, 1.1–8.0, and 1.1–4.2, resp.).

## 4. Discussion

Aspirin, usually available as an over-the-counter drug, is one of the most used drugs worldwide. In Western countries, 6 to 12% of the general population is exposed to low-dose aspirin [15, 16]. It is well known that aspirin use is associated with upper gastrointestinal adverse effects [17]. However, only a small proportion of low-dose aspirin users develop peptic ulcer or life-threatening ulcer bleeding. Currently, the main genetic risk factors determining the development of peptic ulcer in low-dose aspirin remain unclear and pose a fascinating challenge in gastroenterology. In the current study, we investigate the clinical and genetic factors related to the development of peptic ulcer in low-dose aspirin users. The data indicated that the blood type O, advanced age, history of peptic ulcer, and use of NSAID were independent factors predictive of ulcer development in low-dose aspirin users. The C-1676T polymorphism in the* COX-1* gene promoter is not a risk factor for ulcer formation during treatment with low-dose aspirin.

Blood group O has traditionally been associated with the risk of developing peptic ulcer [18, 19]. However, it remains unclear whether a subject with blood group O is a genetic marker for ulcer development during low-dose aspirin use. In this study, peptic ulcer group had a higher rate of blood type O than that of the control group (45.9% versus 29.4%). Multivariate analysis with logistic regression documented that blood type O was an independent factor for ulcer development in low-dose aspirin users with an odds ratio of 2.1 (95% CI: 1.1–4.2). Our study is the first in identifying blood group O as a genetic risk factor of peptic ulcer formation during treatment with low-dose aspirin.

Cyclooxygenases catalyze the conversion of arachidonic acid to prostaglandins. As a corollary,* COX* gene polymorphisms could be important in the pathogenesis of peptic ulcer disease because the function of cyclooxygenases affects prostaglandin formation and its protective effect at the level of the gastric mucosa. A previous study showed that the A-842G polymorphism did not play a significant role in the development of ulcer in NSAID users in the Japanese population [11]. In this study, we investigated the association between genetic polymorphisms in the* COX-1* gene promoter and peptic ulcer development in low-dose aspirin users. Our data indicated that no significant differences in genotype frequencies of the C-1676T polymorphism in the* COX-1* gene promoter were found between the peptic ulcer group and control group. However, Arisawa et al. reported that the C-1676T functional polymorphism in the* COX-1* gene promoter was related to the development of NSAID-induced ulcer [11]. The reasons for the contradictory results are unclear, but different doses of NSAIDs (low-dose aspirin for cardiovascular protection versus regular-dose NSAIDs for various neuromuscular diseases) or different ethnic populations are possible explanations.

In this study, we confirmed that advanced age, history of peptic ulcer, and use of NSAID were independent factors predictive of ulcer development in low-dose aspirin users. History of peptic ulcer is the most important clinical risk factor for ulcer development in low-dose aspirin use with an odds ratio of 27.6. The finding was consistent with previous studies that also identified a history of peptic ulcer as a clinical risk factor for peptic ulcer in low-dose aspirin users [20, 21]. PPI has been shown to effectively reduce the risk of developing peptic ulcers associated with the continuous use of low-dose aspirin [22, 23]. Therefore, cotherapy with a PPI to prevent the development of ulcers and ulcer complications could be considered in the aged patients with a previous history of peptic ulcer [24, 25].

Several other independent studies [26, 27] have also highlighted the importance of age factor in ulcer formation. García-Rodríguez and Jick [26] proved that advanced age increases risk of ulcer complications in an NSAID user. Lanas and Scheiman [4] also demonstrated that advanced age was an independent risk factor of ulcer formation in low-dose aspirin users. The reason why the aged stomach is more vulnerable to injury remains unclear. However, aging-related changes in gastric mucosa defense are possible explanations. Two human studies [28, 29] have demonstrated that gastric mucosal prostaglandin content declines with age. Feldman and Cryer [28] have also disclosed that advanced age is associated with a significant decline in gastric bicarbonate, sodium ion, and nonparietal fluid secretion. Thus, aging is associated with selective and specific changes in the gastric mucosal defenses that may predispose aged aspirin users to develop peptic ulceration.

In the current study,* H. pylori* infection was detected in 26.2% of peptic ulcer subjects and in 47.5% of the controls. The infection rate was significantly lower in the peptic ulcer group than in the control group. The reason for the lower* H. pylori* infection rate in the peptic ulcer group was probably due to prior anti-*H. pylori* therapy in many aspirin users in this patient group. Among the patients in peptic ulcer group, 50.5% of the subjects had a history of prior peptic ulcer. Multivariate analysis indicated that the history of peptic ulcer was the independent risk factor for the development of peptic ulcer and* H. pylori* status was removed from independent factors related to peptic ulcer development following logistic regression analysis.

The strengths of this study included investigating both clinical and genetic risk factors for ulcer development in low-dose aspirin users and prospective assessment of clinical data of the recruited subjects. However, it has several limitations. First, the sample size was moderate and possibly unable to detect minor independent risk factors for the development of peptic ulcer in low-dose aspirin users. Secondly, aspirin users who took PPIs or histamine-2 receptor antagonists before endoscopy were excluded. We therefore could not identify some protective factors for ulcer development in low-dose aspirin users. Thirdly, it was a case-control study and selection bias of recruited cases could not be completely ruled out. A large-scale, long-term cohort study is therefore merited to clarify the risk factors identified in this work.

In conclusion, blood type O, advanced age, history of peptic ulcer, and use of nonaspirin NSAID are independent risk factors for development of peptic ulcer in low-dose aspirin users. The C-1676T polymorphism in the* COX-1* gene promoter is not a risk factor for ulcer formation during treatment with low-dose aspirin.

## Figures and Tables

**Table 1:** Clinical characteristics of aspirin users with and without peptic ulcer.

	Gastritis/normal (*N* = 109)	Peptic ulcer (*N* = 111)	*P* value
Age	67.0 ± 12.1	72.0 ± 10.3	0.001*
Sex			0.501
Female	40 (36.7%)	31 (27.9%)
Male	69 (63.3%)	80 (72.1%)
Cigarette smoking	13 (11.9%)	19 (17.1%)	0.275
Heavy drinker	7 (6.4%)	9 (8.1%)	0.630
Coffee consumption	22 (20.2%)	11 (9.9%)	0.033*
Tea consumption	36 (33.0%)	27 (24.3%)	0.153
History of peptic ulcer	4 (3.7%)	56 (50.5%)	0.000*
Dual antiplatelet therapy	10 (9.2%)	10 (9.0%)	0.966
Coumadin use	1 (0.9%)	3 (2.7%)	0.622
Nonaspirin NSAID use	7 (6.4%)	18 (16.2%)	0.022*
Steroid use	2 (1.8%)	2 (1.8%)	1.000
*H. pylori* infection	52 (47.7%)	29 (26.1%)	0.005*

*denotes *P* < 0.05.

**Table 2:** Blood group and *COX-1* genotype in low-dose aspirin users with and without peptic ulcer.

	Gastritis/normal	Peptic ulcer	*P* value
Blood type	(*n* = 109)	(*n* = 111)	0.047
A	36 (33.0%)	29 (26.1%)
B	38 (34.9%)	25 (22.5%)
O	32 (29.4%)	51 (45.9%)
AB	5 (4.6%)	6 (5.4%)
*COX-1* genotype∗	(*n* = 91)	(*n* = 95)	0.245
T/T	15 (19.5%)	22 (24.7%)
C/T	59 (76.6%)	59 (66.3%)
C/C	3 (3.9%)	8 (9.0%)

*One hundred and eighty six DNA samples were available.

**Table 3:** Multivariate analysis for clinical and genetic factors related to the development of peptic ulcer in aspirin users.

Clinical factor	Coefficient	Standard error	Odds ratio (95% CI)	*P* value
Advanced age	1.248	0.464	3.1 (1.4–8.6)	0.007
Peptic ulcer history	3.320	0.576	27.6 (8.9–85.5)	<0.001
Nonaspirin NSAID use	1.154	0.525	2.9 (1.1–8.0)	0.045
Blood type O	0.751	0.351	2.1 (1.1–4.2)	0.032
